# Information, regulation and coordination: realist analysis of the efforts of community health committees to limit informal health care providers in Nigeria

**DOI:** 10.1186/s13561-016-0131-5

**Published:** 2016-11-14

**Authors:** Seye Abimbola, Kemi Ogunsina, Augustina N. Charles-Okoli, Joel Negin, Alexandra L. Martiniuk, Stephen Jan

**Affiliations:** 1 School of Public Health, Sydney Medical School, University of Sydney, Rm 128C, Edward Ford Building A27, Sydney, NSW 2006 Australia; 2 National Primary Health Care Development Agency, Abuja, FCT Nigeria; 3 The George Institute for Global Health, Sydney, NSW Australia; 4 Department of Epidemiology, University of Alabama at Birmingham, Birmingham, AL USA; 5 Department of Community Medicine, University of Nigeria Teaching Hospital, Ituku-Ozalla, Enugu Nigeria; 6 Dalla Lana School of Public Health, University of Toronto, Toronto, ON Canada

**Keywords:** Informal providers, Health care markets, Community health committees, Primary health care, Transaction costs, Governance, Nigeria

## Abstract

One of the consequences of ineffective governments is that they leave space for unlicensed and unregulated informal providers without formal training to deliver a large proportion of health services. Without institutions that facilitate appropriate health care transactions, patients tend to navigate health care markets from one inappropriate provider to another, receiving sub-optimal care, before they find appropriate providers; all the while incurring personal transaction costs. But the top-down interventions to address this barrier to accessing care are hampered by weak governments, as informal providers are entrenched in communities. To explore the role that communities could play in limiting informal providers, we applied the transaction costs theory of the firm which predicts that economic agents tend to organise production within firms when the costs of coordinating exchange through the market are greater than within a firm. In a realist analysis of qualitative data from Nigeria, we found that community health committees sometimes seek to limit informal providers in a manner that is consistent with the transaction costs theory of the firm. The committees deal not through legal sanction but by subtle influence and persuasion in a slow and faltering process of institutional change, leveraging the authority and resources available within their community, and from governments and NGOs. First, they provide information to reduce the market share controlled by informal providers, and then regulation to keep informal providers at bay while making the formal provider more competitive. When these efforts are ineffective or insufficient, committees are faced with a “make-or-buy” decision. The “make” decision involves coordination to co-produce formal health services and facilitate referrals from informal to formal providers. What sometimes results is a quasi-firm—informal and formal providers are networked in a single but loose production unit. These findings suggest that efforts to limit informal providers should seek to, among other things, augment existing community responses.

## Background

Informal health care providers are typically unregistered and unregulated entrepreneurs operating in local health care markets without formally recognised health care training, although they may have received some informal training through apprenticeships [1]. One of the consequences of ineffective governments in many low- and middle-income countries is that they leave space for informal providers to deliver a large proportion of health services [2, 3]. This is the case in the health care markets within the communities in many low- and middle-income countries. Users of health services in such settings may have difficulty in obtaining, interpreting and using credible information [4]. In addition, regulation of the health care market may be ineffective due to the limited capacity (technical and financial) and incentives to monitor and enforce the rules governing the demand and supply of health services [3, 5]. These lead to a situation in which patients tend to shop around, receiving sub-optimal or inappropriate care and incurring costs and complications until they eventually (if they do) find the appropriate health care provider who is able to successfully manage their condition [2, 6–8]

Informal providers include drug sellers or chemists, traditional healers, traditional birth attendants, and village doctors. Their practice may be herbal or based on modern medicine, and may also be general or specific for procedures such as deliveries or bone-setting [1]. Informal providers thrive within local health care markets without institutions that facilitate appropriate health care transactions [2, 8–10]. The prevalence of informal providers varies widely from one community and country to another—from 88% and 96% of all providers in some communities in Bangladesh [11, 12] to 77% in Uganda [13] and between 51% and 55% in India [14, 15]. The uptake of their services also varies—for drug sellers it ranges from 62% for diarrhoea in Uganda [16], to between 36% and 50% to treat fever in Nigeria [17–20], and 35% for sexually transmitted infections in Uganda [21]. In Bangladesh, 52% to 60% of consultations are with different categories of informal providers [22], 56% in India [23] and 71% in Nigeria [7]. In Uganda, 40% receive treatment for diarrhoea from traditional healers [16] and in Mozambique 43% of pregnant women deliver using traditional birth attendants [24].

Addressing the presence of and preference for informal health care providers in local health care markets requires effective governance [25]. In 2007, due to concerns that the presence of informal providers in local health care markets was a potential barrier to accessing formal providers, the government of Malawi imposed a ban on traditional birth attendants, as a strategy to reduce maternal deaths. Within a year, there was about 15% shift (from 43% to 28%) in the communities where the use of traditional birth attendants was highest at baseline – 11% to formal providers and 4% to home deliveries assisted by relatives and friends. Many pregnant women continued to use the services of traditional birth attendants albeit illegally. The ban was controversial and unpopular, it did not result in improved health outcomes, and was lifted in 2010 [26]. More commonly proposed policy responses to limit informal providers in low- and middle-income countries include government intervention to enforce existing regulation, provide training to improve their services, foster relationships between them and formal providers, and reduce demand for their services by improving access to formal providers [1].

On the other hand, support for community-led initiatives has not been considered as a strategy for reducing the presence of and preference for informal providers [1, 2]. Community engagement in primary health care (PHC) has been extensively promoted in many low- and middle-income countries by establishing community health committees which are linked to the public sector health facility (typically the only formal health service provider) in the community. These community structures have been established across many low- and middle-income countries [27]. Studies show that the activities of community health committees can lead to improvements in the demand and supply of formal health services in their community [27, 28]. In addition, community engagement can move people from being passive consumers to more active roles in service delivery, and in co-financing, co-managing, and co-producing health services [28, 29].

In line with the national health policy, each community in Nigeria is expected to have a community health committee [30]. Typically formed through a participatory approach, national guidelines specify that committee members may include ‘respectable’ members of the community, the health worker in charge of the health facility to which the committee is linked, representatives of traditional, voluntary, religious, women, youth, and non-health occupational groups (primary and secondary school head teachers, and workers in the electricity and water sectors), and notably, representatives of informal health care providers such as traditional healers, traditional birth attendants and patent medicine vendors [31, 32]. Thus, implicit in the design of the committee in Nigeria is the intent that they will influence informal providers, although the guidelines are not explicit about the details of how, under what circumstances and to what end. Understanding these details is important for improving how the committees may be supported to address the challenge of informal health care providers [29, 33]. We conducted a qualitative study in Nigeria to understand how and under what circumstances community health committees seek to limit informal providers operating in their local health care market.

## Methods

### Data collection

The study was conducted between November 2014 and January 2015 in four (out of the 36) states in Nigeria: one in northern Nigeria (Kaduna), two in central Nigeria (Nasarawa and Benue) and one in southern Nigeria (Lagos). The states were chosen for their geographic and ethnic spread. Each of the states has an average of about 20 local government areas. Two local government areas were randomly selected in each state, and from each local government area, two communities were purposively selected, for having a health committee. In all, 16 communities were included in the study. For the in-depth interviews, four categories of study participants were purposively selected based on their availability, on granting informed consent to participate, and on their potential to provide rich, relevant and diverse information: (1) the health worker in charge of the health facility and/or the longest-serving health worker at the facility; (2) the officials of the community health committees, such as the chairman, secretary and treasurer; (3) community members, i.e. individuals in the community who are not members of the health committee, such as religious, women’s and traditional leaders; and (4) primary health care managers employed by the federal, state and local government levels, who provide support for primary health care in the selected communities. However, for the focus group discussions, we included all committee members who were available, had not participated in the in-depth interviews, and gave consent to participate. We excluded potential participants who were less than 18 years old.

We developed semi-structured questions and prompts to explore issues affecting the supply and demand of primary health care services in the communities. If the respondents cited issues related to informal health care providers, the study instrument provided scope to probe how and why and efforts to limit the influence of informal health care providers. The questions included: (1) What are the challenges of health service delivery in this community (or communities)? (2) What other health care providers are in the community (or communities) besides the health facility? (3) What do people in this community (or communities) do when someone becomes ill? (4) Who do you (or people in communities) trust for information about health and health care? (5) What would make people in this community (or in communities) who don’t more likely to use the health facility? (6) Who influences people in this community (or in communities) to use or not use particular health services in and how? (7) What are your (or committee members’) reasons for joining the committee? (8) What are your (or committee members’) challenges discharging responsibilities in the committee? (9) What organisations (government and non-government) does the committee (or committees) work with and how? Notably, the part of the questions in brackets were used during interviews with primary health care managers to apply more broadly to the local government area or state where they work.

We conducted one focus group discussion with health committee members in each of the 16 communities included in this study. Recruitment for in-depth interviews continued until saturation of themes was achieved. In all, we conducted 130 in-depth interviews: with 25 PHC workers (of which 14 were health facility officers in charge), and with 15 PHC managers working at the local (7), state (4) and federal (4) tiers of government. We also interviewed an average of 1 to 2 officials of each committee (making 29 interviews), 2 to 3 other members of each committee (making 35 interviews) and 1 to 2 people in each community who are not committee members (making 26 interviews). Each interview lasted about 60 min and each group discussion involved 8 to 10 people and lasted about 90 min. Data were collected by eight trained researchers in pairs; one pair in each state over the period of about three months. Researchers with postgraduate degrees in public health were selected for their ability to speak the local languages of their respective study states. They were briefed for the purpose of this study by two of the authors (SA and KO). Researchers and participants met for the first time during the study, but there were prior telephone contacts to schedule data collection. Interviews and discussions were conducted within health facility premises or an open space nearby. Researchers kept notes during the field work and were debriefed by author SA at the end of data collection. When required, the data were translated to English by the researchers who collected the data. The interviews and group discussions were audio-recorded, and subsequently transcribed to aid analysis.

### Data analysis

In line with the practice of using the small sample case study design to investigate informal institutions (considering the logistics, time and cost constraints of collecting in-depth large sample data on collective action), the study was designed as a comparative case study [34, 35]. Each community is the unit of analysis. We conducted directed content data analysis by coding and categorising patterns in the data based on an existing framework [36]: two authors (SA and AC-O) read the transcripts and independently reframed data from each community based on the context-mechanism-outcome framework used in realist analysis [37]. This is an approach to theory-driven inquiry; an interpretive framework for hypothesis formulation, data collection, analysis and synthesis, and for testing and refining theories to explain how complex social programmes work. The aim of our analysis was not to determine whether or not efforts of community health committees “work” in limiting informal health providers, but to explore how the efforts were influenced, enabled, and constrained by the interactions between contextual circumstances and the reasoning of the actors involved. The realist approach accepts the role of actors in change (i.e. agency), and that while structural (and institutional) features may exist independent of actors, they influence actors’ individual and group choices and patterns of behaviour. Given this ontological perspective, we sought to unearth social layers (i.e. agency and structure) in order to understand the phenomenon under inquiry; i.e. community efforts to limit informal health providers. While the findings in this study may not necessarily be generalisable to other states in Nigeria and other low- and middle-income countries, the context-mechanism-outcome (CMO) configurations which result from realist analyses can facilitate the transferability of insights to other settings. This is because these CMO configurations can be tested and refined with empirical data in other settings [37].

For this realist analysis, we adapted the stepwise approach to realist analysis proposed by Danermark et al [38], in four steps. In the first step, we identified from the transcripts, events related to limiting the use of informal providers that occurred as a result of committee actions, decisions or relations. These were coded as *outcomes*. In the second step, we identified factors related to the community or committee that either enabled or constrained the outcomes (i.e. *contexts*). The list of outcomes and context expanded as coding proceeded, and they were debated, refined and adjusted between two authors (SA and AC-O). Disagreements in coding and discrepancies in interpretation were decided by consensus among the authors, with insights from the researchers who collected the data, and in consultation with PHC managers in Nigeria.

**Fig. 1:**
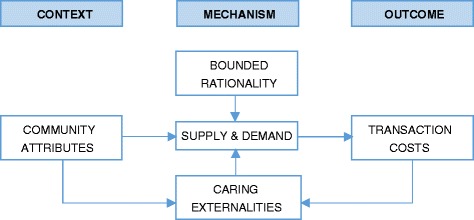
Conceptual framework that informed data analysis, incorporating the theories of bounded rationality, caring externalities and transaction costs. Note: the initial level of transaction costs is part of the attributes of a community, i.e. context

In the third step, we iteratively reframed the identified outcomes and context within theories in order to aid our understanding of the phenomenon under investigation. We adopted the transaction costs theory of the firm which predicts that economic agents tend to organise production within firms when the costs of coordinating exchange through the market is greater than within a firm [39]. This conceptual framing was informed by aspects of the data which support the following two theoretical propositions (see Fig. 1):

1. Humans are boundedly rational economic agents [40]. In this study, we define bounded rationality as a situation in which users and potential users of health services in a community are limited in their abilities to identify appropriate health care providers. In health care markets, consumers are limited by the quantity and quality of the information they have, their own cognitive limitations and the amount of time they have to make the decision about which health care provider to use or not use. Therefore, rather than searching for the optimal health care provider, consumers “satisfice”: they choose the first available alternative that “satisfies” what they perceive to be the minimum criteria for addressing their health needs [41]. This leads to many consumers incurring costs and complications from one provider to another before the appropriate patient-provider transaction, which increases transaction costs of accessing health care. The information asymmetry between service users and providers that results from bounded rationality is predictably worse in under-governed communities with contextual attributes (e.g. geographical or socio-economic) that allow for the presence of unregulated providers [42]. The transaction costs of accessing health care in such a community will increase with increasing number of such providers in the local health care market.
2. However, people care about the health of others and their ability to access health care services. This is described in economic terms as the “caring externalities” of health care markets. In other words, the social benefit of health care exceeds private benefit [43]. Therefore, individuals with relatively higher capacity to distinguish providers within the local health care market based on expected or experienced quality of services, will have a tendency to contribute (if they are able, given the context) towards reducing the transaction costs of accessing health care for people in their community. When the transaction costs of accessing health care are sufficiently high, such individuals will seek to change the institutions governing the demand and supply of health care services in their community; from governance through the price mechanism in the local health care market, to network or collective governance, in which community representatives, through the community health committee, take an active role in the local health care market to reduce the transaction costs of accessing health care [44]. The local health care market becomes a “quasi-firm”, consisting of the people and health care provider(s) working together as a unified production unit of health care services in a community.

In the fourth step, informed by these theoretical propositions, we sought to identify the reasoning process (i.e. *mechanisms*) that led to the outcomes, in line with the findings of an earlier study on how community health committees function in Nigeria [29]—see Table 1.

**Table 1:** Modes of functioning of commmunity health committees in Nigeria

The mechanisms that informed outcome strategies to limit informal health providers were coded as:
**▪ ‘Village Square’** (Mode I) — if the outcome was achieved by the committee using meetings and the personal network of individual members to limit informal providers in their community;
**▪‘Community Connectors’** (Mode II) — if the outcome was achieved as committee members connect the voice of one category of health system actors to ears of other health system actors in the community;
**▪‘Government Botherers’** (Mode III) — if the outcome was achieved by committee members connecting the voice of community members and health workers to the ears of government officials;
**▪ ‘Back-up Government’** (Mode IV) — if the outcome was achieved by committees acting as back-up to the government; co-financing or co-managing health services with governments or NGOs;
**▪ ‘General Overseers’** (Mode V) — if the outcome was achieved by the committee positioning itself as overseers of the day to day running of health services, and financing health care through user charges.

Source: Abimbola et al. [29]

### Ethics

Ethics approval for this study was provided by the National Health Research Ethics Committee of Nigeria. Participation was voluntary, based on the participant signing a written informed consent form. In line with the terms of consent to which participants agreed, the data for this study are not publicly available and all participants have been de-identified, by removing potentially identifying information such as name, gender, cadre, community and local government area of participants. The de-identified data are available on request, but in reporting the findings, we included superscripts linked to a combination of letters and numbers (see Endnotes) referring to communities or PHC managers when the context, mechanism or outcome being reported occurred, was identified or described in that community or by the PHC manager. We used the first letter of each state to denote the state, the letters A and B to denote the two local government areas in each state, and the numbers 1 and 2 to denote the communities in each local government area. A finding from community number 1 in local government A in Benue is marked BA1. When reference is being made to a local government PHC manager, it is to the first letter of the state, and the local government whether A or B; e.g. BA or BB. When reference is being made to a state PHC manager, the letter S is used in combination with the first letter of the state; e.g. BS. When reference is being made to a federal PHC manager, the letter F is used in combination with the first letter of the state in which the federal PHC manager is based; e.g. BF.

## Results

**Fig. 2:**
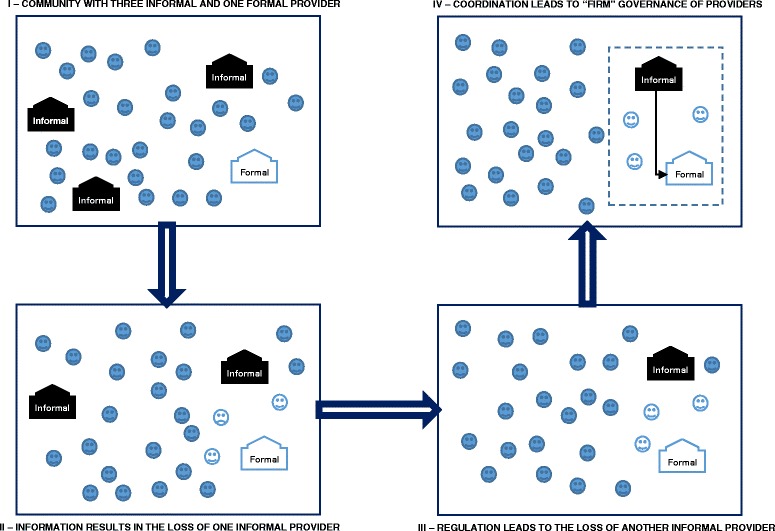
Four stylised schematic depictions representing how community health committees may respond to informal health care providers. Note: blue smiley faces represent community members; white smiley faces represent community members who became members of the community health committee

We obtained information from 223 community health committee members; 64 of whom participated in the interviews and 159 in the discussions. Of the participants who were community health committee members, there were 40 in Lagos, 43 in Benue, 77 in Nasarawa and 63 in Kaduna. Their average age ranged from 43 years in Kaduna to 49 years in Lagos, with overall average age of 46 years. The male to female distribution in Lagos was 1:1, whereas it was 2:1 in Benue, Nasarawa and Kaduna. Lagos had the highest proportion of committee members with tertiary education, with 50% having attained some form of tertiary education. Further, 80% of committee members in Lagos had a dependable income either as business owners or civil servants, compared to between 38% (Benue) and 57% (Nasarawa and Kaduna). In Benue, 62% of committee members were either unemployed or subsistence farmers with no ready cash income, while 43% of committee members in Nasarawa and Kaduna were in this category, but only 18% in Lagos. Based on the interviews, discussions and observations, we identified three categories of events resulting from the actions, decisions and relations of community health committee members which may influence the use of informal health care providers: information, regulation and coordination – see Table 2 and Fig. 2.

**Table 2:** How community health committees limit informal health care providers in Nigeria

Outcome (Outcome Strategies)	Mechanism (Modes of Functioning)	Context (Enablers and Constraints)
*Information* Formal – Encouraging the use of formal services at the health facility through personal contact and campaigns Informal – Discouraging the use of informal health care providers community through personal contact	Mode I: Village Square Mode II: Community Connectors Mode III: Government Botherers Mode IV: Back-up Government Mode V: General Overseers * Triggered by need to reduce the transaction costs of accessing health care	■ Having the autonomy to modify membership to have committee members with rich personal network and wide reach in the community. ■ Significant health events like disease outbreaks and vaccine refusal and support to conduct information campaigns from governments, NGOs and traditional leaders. ■ High cost of participation in meetings and information campaigns in large communities and where members cannot afford the cost of transportation. ■ The extent of competition from informal providers in the local health care market – low levels of competition removes necessity for information campaigns.
*Regulation* Formal – Monitoring formal health service delivery to ensure responsiveness, quality and credibility Informal – Monitoring informal health care providers to keep their activities within safe limits	Mode I: Village Square Mode II: Community Connectors Mode III: Government Botherers Mode IV: Back-up Government Mode V: General Overseers * Triggered by need to reduce the transaction costs of accessing health care	■ Having responsive government PHC managers who discipline health workers or transfer them elsewhere at the behest of committee members. ■ Traditional leaders who admonish health workers or facilitate the link of committees to government PHC managers to effect behaviour change among health workers. ■ More challenging to monitor and regulate informal providers out of the market when they are many and control a large share of the local health care market. ■ Mentoring by NGOs to facilitate monitoring of traditional birth attendants and inviting them provide services in the health facility to enhance monitoring.
*Coordination* Formal—Mobilising resources to improve the quality and accessibility of formal services at the health facility Informal—Facilitating referral from informal to formal health care providers in the community	Mode I: Village Square Mode II: Community Connectors Mode III: Government Botherers Mode IV: Back-up Government Mode V: General Overseers * Triggered by need to reduce the transaction costs of accessing health care	■ Having high income people on the committee and in the community who rely on the health facility, else committees need traditional leaders to help raise funds from them. ■ Highly networked communities where committee members belong to other community groups helps fund-raising from religious, women’s, youth and cultural groups. ■ Mentoring on fund raising by and donations from NGOs and traditional leaders; and mentoring on fund raising from government PHC managers. ■ Having traditional leaders, women’s groups and NGOs that help committees to broker agreements between informal providers and the health facility.

Context–mechanism–outcome (CMO) configurations explaining how community health committees limit informal health care providers in Nigeria

Source: findings of this study

### Information

The idea of having a community health committee was typically introduced to communities by government PHC managers^1^ and NGO representatives.^2^ But the idea also arose spontaneously,^3^ triggered by concerns about the challenges experienced and costs incurred by people in accessing health care. In such settings, the founding mission of the committees included self-support due to government neglect^4^ and a commitment to reduce maternal and child deaths by stopping women from delivering at home unattended or by traditional birth attendants.^5^ The founding mission also included wanting to improve the uptake of formal health services^6^ Even when not spontaneous, communities see having such a committee as an opportunity to address health care challenges in the community, motivated by the altruistic desire to see more community members use existing formal health services in the community.^7^ Essentially, committee members say to the community: “the health facility is there, cheaper^8^ and of better quality^9^ than the alternatives, and put in place by your government, so please use it; it’s your right,^10^ take advantage of the opportunity.”

However, committee messaging depends on which provider is the major competition. Where the public sector health facility is the only formal health provider in the community, the emphasis is on the health facility being of better quality than its competition.^11^ But what is emphasised in Lagos where there are private sector formal health providers is that the health facility is cheaper than its alternatives^12^ or that it is the only health facility that runs 24 h services.^13^ When chemists are the competition, members advise people of the dangers of self-medication with drugs from chemists (e.g. more likely to be fake, expired or wrongly dispensed) and they emphasise the lower cost of drugs at the health facility.^14^ When traditional healers and birth attendants are the competition, committees emphasise the downsides of consulting them by citing instances of bungled cases and their lack of skills and diagnostic tests compared to the health facility.^15^ But where traditional providers are not a major competition, there is less drive to displace them from the market.^16^

Functioning in the ‘village square’ mode, the majority of committees meet monthly and spread information about services at the health facility through their family and friends.^17^ Committees expand their membership to include individuals with rich personal network—pastors and imams who can reach out to their congregation,^18^ women and youth leaders^19^ and people who belong to other community groups.^20^ But realising that they need to do more to increase the uptake of formal health services, committees position themselves as ‘community connectors’, conducting information campaigns,^21^ and providing information on services in the health facility in religious gatherings. 22They also spread information through weddings and naming ceremonies,^23^ community meetings,^24^ and house to house visitations. 25Committees use town criers to announce clinic schedules, community outreaches and information campaigns.^26^ Committees also count on the word of mouth of people who have used the health facility, tried informal providers and discovered how high the quality of services at the health facility is relative to informal providers.^27^ Where cultural beliefs underlie the use of traditional providers, committees resort to messaging directly from traditional leaders.^28^

By spreading information through these channels, committee members and other community members, health facility officers in charge and government PHC managers, report significant increases in the use of the health facility and reduction in the use of informal providers.^29^ In some communities, particularly in Lagos, the use of information is so successful that it displaced traditional healers and birth attendants from the local health care market.^30^ However, the costs of participation in information campaigns can be overwhelming for committee members who are unemployed or are subsistence farmers who need to take time off their work for meetings and campaigns,^31^ or in large communities with poor access roads. 32In such situations, committees resort to other mechanisms to ensure they conduct information campaigns—as ‘government botherers’ they lobby the government to support campaigns,^33^ as ‘back-up government’ they raise funds from NGOs and the community to support campaigns^34^ and as ‘general overseers’ they use health facility revenue to fund campaigns.^35^

However, in spite of high costs of participation, some committee members reckon that the benefits they derive from participation outweigh the costs^36^; benefits such as the dignity that comes with community recognition and gratitude^37^; knowledge from the training they receive to help them spread health information^38^; and being treated at reduced prices when they and their family use the health facility. 39But some committee members expect financial incentives whether or not participation for them is costly, and they tend to resign when these are not forthcoming.^40^ Such expectations are higher where NGOs have once provided financial incentives^41^; in neighbouring communities where committee members wonder why they are not enjoying the same benefits; and where community members are less willing to contribute funds to support committee members even after payments are discontinued.^42^ Nonetheless, the majority of committee members work voluntarily without financial reward, and they encourage others to look beyond financial benefit.^43^

Contextual enablers of the information strategy include the autonomy of committees to decide who to invite to meetings and membership.^44^ For example, to improve their information campaigns, committees invite as members people who are outspoken, popular and can pull crowds.^45^ Training provided by governments and NGOs on how to conduct information campaigns is another enabler of the information strategy.^46^ Events such as disease outbreaks and vaccine refusal trigger information campaigns by the committees. 47Government funding for information campaigns is an enabler of the information strategy, with government PHC managers using the occasional opportunity of funded campaigns to provide financial incentives to committee members.^48^ But lack of competition within the local health care market makes the information strategy less necessary. In some communities, this happens only after an initial period in which committees have successfully displaced their competition from the health care market. Such committees meet or hold information campaigns less frequently, especially where the cost of meetings and campaigns is particularly high.^49^

### Regulation

Committees also engage in activities to ensure the responsiveness, quality and credibility of the health facility to which the committee is linked, by monitoring and supervising the health facility and health workers. On the other hand, the committees also seek to keep the activities of informal health care providers within safe limits. Where information has been particularly effective, informal providers self-regulate or exit the local health care market.^50^ To displace informal providers from the market, it is often necessary to build trust in the quality and credibility of the health facility. But in spite of information on which provider is appropriate, people may not use formal health services because of disrespectful and abusive treatment,^51^ health worker absenteeism^52^ and unreasonably high cost of medicines and services.^53^ Working as ‘village square’, committees obtain these feedback during general community meetings,^54^ and when non-members attend committee meetings to discuss the challenges they experience while using the health facility.^55^ People also give feedback during information campaigns when committees are functioning as ‘community connectors’.^56^

Functioning in the ‘back-up government’ and ‘general overseers’ modes, committee members ask health facility officers in charge about inappropriate behaviour of health workers,^57^ and they also meet with people individually to find out what will increase their preference for the health facility.^58^ Committee members visit the health facility, observe health workers as they deliver services, listen to the complaints of people accessing care and intervene immediately or during their monthly meetings.^59^ Usually once a week, committee members visit the health facility unannounced to check absenteeism.^60^ To ensure that the health facility can outcompete other providers in the health care market, committees set the price medicines and services accordingly,^61^ a strategy that is enabled where committees operate government-established revolving funds for drugs and service uptake is sufficient to afford such price flexibility.^62^ In addition, committee members visit the health facility to ensure that people are not being overcharged^63^; and also ask people how much they buy drugs in the health facility to ensure it is according to government price schedules.^64^

However, committees achieve the outcome of respectful service delivery, appropriate prices and reduced absenteeism^65^; especially where, as ‘government botherers’, there is recourse to responsive government PHC managers who are willing to exercise a strong hand in disciplining health workers^66^ or having them transferred elsewhere.^67^ These outcomes are constrained where committees do not enjoy good relations with government PHC managers.^68^ But even with unresponsive government PHC managers, these outcomes are still achieved where traditional leaders admonish health workers or facilitate their link to government decision-makers.^69^ In addition, committees lobby to reverse the transfer of high-performing health workers.^70^ They also convince health workers to modify or reorganise service delivery at the health facility to ensure greater uptake; such as creating a first aid outpost to reduce the need for trips to the health facility in a large community,^71^ changing the immunisation and clinic days to the market day so that it is convenient for mothers, 72and allowing pregnant women to deliver lying on the floor as they prefer and are allowed to do by traditional birth attendants.^73^ By these means, committees increase the uptake of formal health services.^74^

Unlike the health facility where there is a recourse to governments as the employer of health workers, it is more challenging to enforce rules governing the supply of health services by informal providers. In this regard, traditional healers and birth attendants enjoy significant advantage: their services are in line with belief systems of many in the community, they offer more flexible payment options, and where they live and work is closer to more people than the health facility.^75^ For this reason, some committees refrain from monitoring them, concerned about their legitimacy to do so^76^; and others because the committee enjoys a referral relationship which they are careful not to ruffle.^77^ However, where there is only one informal provider in a community, committees are sometimes able to ease them out of the local health care market by offering them jobs—in some cases as a cleaner or security guard in the health facility. 78But easing them out of the market is more challenging where they control a larger share of the market due to their sheer number. In these and other communities, committee members monitor traditional healers and birth attendants^79^ Enabled by NGO mentoring, some committees combine monitoring traditional birth attendants with inviting them to bring their clients to the health facility when in labour, so that health workers can directly monitor and mentor the traditional birth attendants as they take deliveries and address complications immediately.^80^

Committees sometimes (as ‘government botherers’) call on government PHC managers to intervene where disagreement and conflict arise when committee members find inappropriate practices as they monitor traditional providers.^81^ But rather than seeking to dislodge traditional providers from the market, committees generally focus on where they have more leverage—they try to make the health facility more desirable.^82^ Compared with traditional providers, it is even more challenging to regulate chemists, who are typically the first port of call for the majority of people, because there can be several of them in a community, and they can be indispensable when health facilities are often out of stock for drugs and supplies.^83^ When they are many, the costs of monitoring and enforcing rules governing the supply of services by informal providers can be challenging, and in such settings committees invest in information strategies instead.^84^ Conversely, where committees are not able to meet and conduct outreaches regularly due to the high costs of transportation, they are still able to monitor the health facility; for those committees, this is a less costly intervention.^85^

### Coordination

The challenges limiting the use of formal health care providers are sometimes beyond what the information and regulation strategies can address. In these instances, the committees seek to coordinate referrals from informal to formal health care providers; and they also seek to coordinate resources from within and outside the community to address the challenges of quality and accessibility limiting the use of the health facility to which the committee is linked. Such challenges are identified by committee members by virtue of living in the community, and as ‘village square’ from their discussions during their committee meetings^86^ or while on monitoring visits to the health facility as ‘general overseers’.^87^ The challenges range from long distance and bad access roads to the health facility^88^; inability to afford the cost of transportation^89^ or the cost of medicines and services^90^; inability to offer 24 h services and long waiting time for patients because health workers are insufficient^91^; health worker absenteeism due to lack of accommodation in the community^92^ and irregular salary^93^; and the health facility being often out of stock for drugs, vaccines and other supplies.^94^

Enabled by having high income community members on the committee, some of whom are invited on the committee for that purpose,^95^ during meetings, committees (as ‘village square’) address some challenges in the health facility requiring petty donations.^96^ But more commonly, committees take their challenges to the government (as ‘government botherers), asking for funds and support to address the issues limiting the uptake of services in the health facility.^97^ In general, governments in Lagos are more responsive.^98^ In Benue and Nasarawa where governments are less responsive,^99^ a low sense of legitimacy,^100^ low expectations due to previous unsuccessful attempts,^101^ and ignorance about the minimum standard of services to expect from their governments^102^ combine to limit the capacity of committees to make demands. However, having community and traditional leaders facilitate lobbying efforts helps to bolster the capacity of committees to relate with governments.^103^ Otherwise, they lobby when government PHC managers visit, or they rely on health facility officers in charge to lobby on behalf of the community, but with poorer results than when members of the committee do the lobbying themselves.^104^

Given their experience and expectation of government failure, committees sometimes lobby governments (as ‘government botherers’) only after they have tried other means of addressing challenges such as raising funds within the community and from NGOs (as ‘back-up government’).^105^ Committees begin to frame their activities as reciprocating government or NGOs support.^106^ They make monthly contributions,^107^ raise funds from other individuals and organisations in the community and from NGOs, and also volunteer their time and labour.^108^ Using their vehicle, committee members, and high income individuals in the community help transport poor people to the health facility,^109^ and also to bring drugs and supplies from the local government headquarters.^110^ To assuage the effects of irregular salary payments, committees provide health workers with food stuff^111^; and to improve formal health service uptake, they volunteer their labour and skills to repair access roads to the health facility.^112^ Committee members make donations so that poor people in the community can use the health facility without paying^113^; and health workers sometimes sell drugs on credit or offer free services on their discretion.^114^ The funds raised are also used to address the reasons why people do not use the health facility.^115^

Raising funds within the community is enabled by having high income people on the committee,^116^ and also having other high income people in the community.^117^ Committees readily receive support when the health facility is the only formal health service provider in and around the community and is thus used by high income community members.^118^ In addition, committees raise funds from local businesses,^119^ politicians,^120^ religious groups,^121^ and other community groups.^122^ But without the help of traditional leaders, raising funds is more challenging when only the poor rely on the health facility and high income people have ready access to alternatives such as a private health facility or general hospital in the community or nearby.^123^ Enabled by the autonomy to determine their membership, committees expand their membership to increase the number of people who make monthly donations.^124^ Government PHC managers, traditional leaders and NGOs mentor committees on fund-raising and sometimes make donations to health facilities through the committees.^125^ To induce further support and community appreciation, committees provide regular report on their revenue and expenditure to the rest of the community.^126^ But when they generate funds from committee members, NGOs and local businesses, they report only to individuals and organisations that support them.^127^

Committees as ‘general overseers’ augment funds raised from the community and NGOs with funds generated from health facility revenue (service charges and medicines). With combined funds from all these sources, committees build or rent accommodation for health workers to reduce absenteeism,^128^ and employ additional health workers as part-time or volunteer staff to address the challenge of not having enough health workers.^129^ Committees derive their sense of autonomy to use health facility revenue by virtue of weak government provision and oversight of health services.^130^ In Lagos where governments are more involved, government PHC managers control health facility revenue.^131^ Elsewhere, health facility officers in charge control the revenue when committees have a low sense of legitimacy or government PHC managers do not trust them to manage the funds accountably.^132^ Committees also derive their sense of legitimacy and autonomy from traditional leaders, who also monitor and require them to provide financial report of health facility revenue to the community.^133^ Accountability for funds among committee members is another contextual enabler.^134^

When efforts to improve services at the health facility are not enough to sufficiently reduce the use of informal providers, committees seek to establish referral networks with informal providers. Traditional birth attendants are encouraged to refer pregnant women who consult them to deliver at the health facility.^135^ When that fails, traditional birth attendants^136^ (and also chemists^137^) are encouraged to refer their clients to the health facility when there are complications or when symptoms fail to subside. But even without complications, traditional birth attendants are still encouraged to bring their patients to the health facility immediately after delivery so that the mother and baby can receive appropriate post-partum care.^138^ However, committees avoid adversarial relations with traditional birth attendants where they are seeking to or have already established a referral relationship with them.^139^ To facilitate networking, traditional birth attendants are invited to meetings,^140^ counselled, and trained with NGO support on danger signs in pregnancy.^141^ Enabled by traditional leaders, members of women’s groups, and NGO officials who help broker agreements with informal providers, the efforts of committees on these referral networks improve service uptake at the health facility.^142^

In addition, committees seek to more directly connect individuals in the community and the health facility by influencing and supporting health workers to offer home delivery services for pregnant women, especially in Kaduna where preference for traditional birth attendants is partly because they offer home services.^143^ Some committee members also actively seek out sick people in the community and refer them to the health facility, based on concerns that otherwise they would resort to self-medication.^144^ But where the contextual enablers for engaging with informal providers are absent and the committee has a low sense of legitimacy to engage with informal providers, they focus instead on improving the supply of services at the health facility.^145^ Efforts of committees to co-produce and so improve the quality and accessibility of formal health services also signal to the rest of the community that the health facility is a collectively owned resource that should be used.^146^ The involvement of traditional and religious leaders, and other influential people in committee activities creates within the community a sense of ownership of the health facility.

## Discussion

The findings from this study reveal that community health committees in Nigeria tend to act as institutional entrepreneurs [45, 46] in response to high transaction costs of navigating local health care markets. They use three categories of strategies (see Table 2): Using information strategies, committees can progressively reduce the share of the market controlled by informal providers, and regulation strategies can keep informal providers at bay while making the formal provider more competitive. When providing information and efforts at regulation are ineffective or insufficient, committees are faced with a “make-or-buy” decision. Depending on the context, some communities are able to adopt the “make” decision by coordinating resources and referrals to improve access to formal providers; they become co-producers of health care while facilitating referral linkages from informal to formal providers. In a response that is consistent with the transaction costs theory of the firm, the network governance structure that emerges from community efforts creates a quasi-firm out of the local health care market [39, 47]; a quasi-firm in which informal providers are networked with typically the only formal provider in a single but loose health care production unit. The committees are therefore active players in the local health care market of their community, acting in a slow and faltering process of institutional change to moderate the influence of informal providers and improve the quality and accessibility of formal providers; dealing not through legal sanction, but with subtle influence and persuasion, and by leveraging the authority and resources available within their community and from governments and NGOs (see Fig. 2).

These findings are in line with the evidence that, whether for altruistic or self-interested reasons, community groups are able to manage their commonly owned resources [9]. Indeed, there are elements in the way in which these committees function which resemble the strategies and circumstances that have been shown to be effective in facilitating community management of common resources. For example, self-organisation is more successful in: 1) small communities where people share common values and trust one another; 2) where people depend on the resource and agree on how to sustain it; 3) where groups use the existing leadership capabilities in the community—involving people who are respected as community leaders and elders, people with prior experience of community organisation, and people of higher socio-economic status; 4) where governments extend autonomy to community groups; 5) where NGOs provide training and resources; and 6) where community groups are large—especially when activities to sustain the common resource are costly and the groups need to raise funds, although larger group size also increases the cost of self-organisation [34, 48, 49]. In addition, our findings are in keeping with extensive experiments on collective action for public goods, which show that people cooperate, albeit sub-optimally, to produce public goods; and that cooperation tends towards being optimal in a small community setting where everyone is aware of everyone else’s ability and level of contribution, than in settings where communication and feedback is more limited [44, 50, 51].

To limit the influence of informal providers within the local health care market, the committees provide institutions of economic governance: securing the property rights of people to maximise the benefits of public goods provided by their governments but derived from their resources and taxes; facilitating appropriate health care transactions while discouraging inappropriate transactions; and encouraging collective action to provide public goods including physical and organisational health care infrastructure [44]. There are examples in the literature of individuals and community groups providing support for health care by enacting different institutions of economic governance, but these activities have not been investigated and explained on their own merit [28, 52–54]. This study suggests that the collective action driven by the committees has underlying motivation in a ‘caring externality’; a situation in which individuals derive benefit from others having access to appropriate health services [43]. In addition, higher income members of the community have their own self-interest in contributing to available health services in their community especially when they depend on the same health facility as others in the community. The other private benefits for committee members is that care at the health facility is often subsidised for them and their family. Further, cultural beliefs also influence committee members: another incentive for participation is the sense of dignity it brings for members as their contributions are recognised by traditional leaders and commended by others in the community.

With government failure (to provide and oversee PHC) and market failure (due to information asymmetry), community groups become a governance alternative [25], as their activities result in a network governance structure bringing together formal and informal providers within the local health care market. It has been previously hypothesised that three levels of governance—governments (constitutional), markets (operational), and community groups (collective)—function in a dynamic balance in which governance failure at one level is assuaged by governance at another level, such that for health care in communities, government failure leads to the dominance of market forces, which may be mediated through governance by community groups [25]. The mechanisms by which community health committees respond to these failures depend on context and the challenge at hand. The findings of this study show that in their efforts to limit informal health care providers, the committees function in all five modes identified in a previous study [29]—as “village square”, “community connectors”, “government botherers”, “back-up government” and as “general overseers”. These five modes of functioning combine in each community to inform the information, regulation and coordination strategies of the community health committees in response to high transaction costs of accessing health care. Bottom-up initiatives are particularly important in low- and middle-income countries due to weaknesses in top-down governance [25], leading to community groups taking on the roles of government as they make, change, monitor and enforce the rules governing the demand and supply of health care in their community.

However, there are constraints on the capabilities of committees to limit informal providers. First is a limited sense of legitimacy to make, change, monitor and enforce the rules governing health care in their community. To gain legitimacy within their community, government PHC managers and NGO representatives who establish, mentor and support the committees should encourage the involvement of influential community members, particularly traditional leaders in selecting committee members and in their day to day activities. Second is the need for financial support from governments and NGOs to hold meetings and information campaigns and to implement projects, due to the high fixed costs of producing health care services. But given that extrinsic rewards tends to undermine intrinsic motivation for high-interest tasks, especially when rewards are tangible, expected, and not performance-based [55], it is important that funds are not presented as rewards or incentives, but for example, as occasional or matching grants. Third is limited legitimacy and power to sanction informal health care providers, demand accountability from formal health care providers and make demands of governments to provide and oversee health care. This requires a mix of strategies, such as having traditional leaders sanction informal providers and make demands of governments and formal providers, providing committees with information and supporting documents on the minimum standards to expect of governments and formal providers to use while making demands, legitimising their authority to demand accountability by enshrining the committees in law, and fostering a democratic culture in which governments are more beholden to communities and their representatives. Committees in Lagos are more effective because it is the state with the most enlightened electorate and effective taxation in Nigeria [56]. Governments elsewhere have weak taxation capacity, and community donations essentially replace taxes.

The perceived cause of an illness (informed by religious or traditional beliefs) and the absence of universal health coverage account in part for why informal health care providers thrive in may low- and middle-income countries. Informal health providers also thrive partly because many instances of illness are either self-limiting or have a low likelihood of treatment complications, such that seeking care from informal providers can be an effective cost-cutting strategy. However, care-seeking pathways can be elaborate for chronic conditions with specific treatment, raising transaction costs. In a previous study in Nigeria, 67% of patients receiving treatment for tuberculosis had earlier consulted chemists and traditional healers, transaction costs accounted for 24% of the costs of care, and 62% of transaction costs were incurred during the first inappropriate consultation [7]. This suggests that interventions should include strategies to limit the costs incurred during the first inappropriate consultation. Further studies are however needed to characterise the transaction costs of access to health care in different populations and for different diseases. But due to cost, logistics and time constraints, we were unable conduct a large sample study, explore the costs of community self-organisation, interview informal providers and collect cost data in this study which may have allowed for more predictive analyses of responses to transaction costs. However, evidence from legislations to ban informal providers from twentieth century Britain [57] and China [58], to contemporary Malawi [26], indicate a slow pace of shifts in preference, which is facilitated by referral linkages from informal to formal providers and “the gradual retirement of the old guard” [57]. Historical studies could investigate institutional change in countries where people have transitioned to near absolute use of formal health services.

## Conclusion

In summary, this study shows that supporting community groups such as the health committees can be an effective policy tool to address the presence of and preference for informal providers in low- and middle-income countries where bottom-up initiatives are important because of the tendency for government weaknesses. In studying and seeking to intervene in such weakly governed health systems, it is therefore important to consider the implications for access to health care of the failure to provide information, regulation and coordination within local health care markets. Indeed, efforts to limit the use of informal providers, whether by governments or NGOs (local and international), should take into account the relative costs of intervening at each level of governance (1. by strengthening the government; 2. by supporting community groups; or 3. by working directly with providers) with the benefits of reducing transaction costs in the community. Having an empirical measure of costs (i.e. transaction costs of accessing health care) against which to compare the costs of interventions to limit informal health care providers can inform the choice among alternative governance interventions. In addition, considerations of potential interventions should take into account existing community responses, the mechanisms through which the community responses are enacted, the contextual enablers and constraints of such responses, the successes, failures and costs of the responses, and to weigh those costs against the cost of intervening at other levels of governance.

## Footnotes

1 (LA1, LA2, LB2, BA2, BB1, BB2, NA2, NB1, NB2, KA1)

2 (BA1)

3 (LB1, NA1, NA2, KA2, KB1, KB2)

4 (LB1, KB2)

5 (KB1, KB2)

6 (NA1, NA2, KA2)

7 (LA1, LA2, LB2, BA1, BB, NB1, NB2, NA, KB1)

8 (LA1, LA2, LB1, LB2, NB1, NB2, KA1, KB2)

9 (LA2, LB1, LB2, NB1, NA1, NB1, KA2, KB1, KB2)

10 (LA1, LB1, NA1)

11 (NB2, KA1, KB2)

12 (LA1, LA2, LB1, LB2)

13 (LA1, LA2, LB2, BB1, KB2)

14 (LB1, NA1, NA2, NB1, KA2, KB1)

15 (BA1, NA1, NA2, NB1, NB2, KA2, KB1, KB2)

16 (LB1, LB2, NA1, NB2)

17 (LA1, LA2, LB1, LB2, BA1, BA2, BB1, BB2, NA1, NA2, NB1, KA1, KA2)

18 (LA2, BA1)

19 (LA2, LB1, BA2, BB2, BB, NB2, KA1, KA2, KB1, KB2)

20 (LB2, BA2, NB1, KA1, KA2, KB1, KB2)

21 (LB2, BA1, BA2, BB1, BB2, NA1, NA2, KB1)

22 (LA2, LB1, LB2, BB1, BB2, NA2, NB1, NB2, KA1, KA2, KB1, KB)

23 (LB1, BA2, KA1, KA2, KB1)

24 (LB2, BA1, BA2)

25 (LB1, LB2, KA2, KB1)

26 (LA1, BB2, NA1, NA2, NB1, KA1, KA2, KB1)

27 (BA1, NA2, NB1)

28 (LB, BA, NA1, NB1)

29 (LA1, LA2, LB1, LA, BA1, NA2, NB1, NB2, NB, NF, KA1, KA2, KB1, KB2, KB)

30 (LA1, LA2, LB1, NB2, KA2)

31 (LA1, LA2, LB1, BA1, BA2, BB2, NA1, NA2)

32 (BA2, BB2, NB1, NB2, KA2)

33 (LA1, LA, LB, NB1, KB)

34 (LB, BA, BB1, BB2, BB, KB1)

35 (BA2, BA, BB2)

36 (LA2, KA2)

37 (LA2, LB, KA1, KB2)

38 (LA1, LB2, BA2, BB2, NA1, NA2, NB, KA1, KA2, KB)

39 (BA1, BA2, NA1, NA2, NB1, KB2)

40 (LS, BB)

41 (BA1, BA2, BB1, BB, NB2, NB, NF, KF)

42 (BA2, BB1, NB2, KF)

43 (LA, LS, BB1, NB1, NB2)

44 (LS, BA1, BA2, BA, KB1)

45 (LB1, LB2, KA2)

46 (LB1, LA, LB, BA1, BA, BB, NA, NB)

47 (BA1, BA2, NB1, NB2, NB, KA1, KB1, KS, KF)

48 (LB1, LB2, LB, NA2, NB1, NB2)

49 (LA1, NA1, NB2, KA2)

50 (LA1, LA2, LB1, NB2)

51 (LA2, LB1, BA1, BB2, NA2, KA1, KB1, KB2, KB)

52 (BA1, BB1, NA2, NB)

53 (NB)

54 (BA2, NB1, KB1, KB2)

55 (BA1, KA1, KA2)

56 (LA1, LA2, LB1, LB2, NA1, KA2)

57 (LB2, NB2)

58 (LB1, BA1, NB2)

59 (LA1, LA2, LB1, LB2, BA1, BA2, NA1, KA1, KA2, KB1, KB2)

60 (BA1, BB1, BB2, NA1, NA2, NB1, NB2, NB, NF, KA1, KA2)

61 (BA1, BA2, BA, NA1, NB, NF)

62 (BB1, BB2, NB1, KB1, KB2)

63 (BA1, BA2, BA, BB1, NA2, NB, KA2, KB1, KB2)

64 (LB, BA, NB2, NF)

65 (LA1, LB1, LB, LS, BA1, BA2, BA, BB2, BB, NA2, NB1, NB2, NS, KA2, KB1, KB2, KB, KS)

66 (LB, LS, NB, NS, KA2, KB1, KB2, KB, KS)

67 (LA1, LA2, LB1, BA1, BA2, BA, BB1, BB2, BB, NB1, NB2, NS, KB1, KB2, KB, KS)

68 (LB2, BB1)

69 (NA1, NB1, KB1)

70 (LB1, BB2, NA1, NB, KA2, KB1, KB2)

71 (LB2)

72 (NA1)

73 (NA1)

74 (LA1, LB1, BA1, KB1)

75 (LA2, LB2, BA2, BB2, NB1, NF)

76 (LB2, BA2, BB1, NA1)

77 (LB2, BA2, BB1, NA1)

78 (BA1, BA2, NB2)

79 (LB1, BA1, BA2, NB1, NB2, KA1, KB1)

80 (BB2, KA1, KA2)

81 (KF)

82 (BB1)

83 (LB2, BA2, BB2)

84 (NA1, NA2, NB2)

85 (LB2, NA1, NB2)

86 (LA1, LA2, LB1, LB2, LB, BA2, BB1, BB2, NA1, NA2, NB1, NB2, KA1, KA2)

87 (LA1, NA1, NB1, KA1)

88 (LA1, LA, LB2, BA2, BB1, BB2, NA1, NB2, KA1, KA2, KB1, KB2, KB)

89 (LA1, BA1, LB2, NA1, KB1, KB2)

90 (LA2, LA, BA1, BA2, BB2, NA1, NB1, NB2, KA1, KB1, KB, KF)

91 (LA2, LA, LB1, LB2, LB, NA1, NB1, KB1, KF)

92 (LA2, LB2, LB, BA2, BB, NA1, NA, KA2, KB1)

93 (BA1, NA1, KB1)

94 (NB1, NB2, KA1)

95 (LB1, KA2, KB2)

96 (LA2, BA1, BA2)

97 (LA1, LA2, LA, LB1, LB2, LB, LS, BA1, BA2, BB1, BB2, NA1, NA2, NB1, NB2, KA1, KA2, KB1, KB2, KB)

98 (LA1, LA2, LA, LB1, LB, LS)

99 (BA1, BA2, BB1, BB2, NA1, NA2, NB1, NB2)

100 (LB2, BA2, BB1)

101 (BA1, BA2, BB1, BB2, NB1, NB2)

102 (BA1, BA2, NB1, NB2)

103 (BA2, NA2, NB1, KA1, KB1, KB)

104 (BA2, BB1, BB, KA1, KB)

105 (LA2, BB, NA1, NB1)

106 (LA2, BA2, NF)

107 (LA2, LS BB2, BB, KA1, KB1)

108 (LB1, LS, BA1, BA2, NB1)

109 (LA1, LA2, LB2, BA2, NA1, NA2, NB1, NB2, NF, KA1, KB1)

110 (NA2, NB2, KA2)

111 (BA1, BB1, NA2, KB1)

112 (BA2, NA2)

113 (LA2, LB1, BA1, BA2, BB2, NA1, NA2, NB1, KA2, KB1, KB2)

114 (NB2, KB2)

115 (LA1, LA2, LA, LB1, LB2, BA1, BA2, BB2, NA1, NA2, NB1, NB2, KA1, KA2, KB1, KB2)

116 (LA2, NA1, KA1)

117 (LA2, BA1,, NA2,, NF, KA2, KB1, KB2)

118 (BB2, NA1, NA2, NB1, KA1)

119 (LA1, LB1)

120 (LA1, LA, LB1,, KB1, KB2)

121 (LA2, BB2, NA2, KA2)

122 (BB2, NA2, NB1, KA1, KB1, KB2, KB)

123 (LA1, LA2, LB2, BA1, BA2, BB1, NA1, NB2, KA1, KB2)

124 (LA1, LB1 LS, BA2, BA)

125 (LB1, LB2, BA2, BA, BB2, NA1, NB1, NB, NS, KF)

126 (LB2, BA1, KA2, KB1)

127 (LA1, LA2, LB1, LA, BB2, KA1)

128 (LB, BA1, BA2, BB2, BB, NA1, NA, NB2, KA2, KB1, KB2)

129 (LA2, BA1, NA2, NB1, NB, KB2)

130 (BA1, BA2, BA, BB2, BB, NA2, NA, NB2, NB)

131 (LA1, LA, LB)

132 (BB1, BB, KA1, KB)

133 (LB1, LB2, BA2, BB2, NA1, NA2, NB1, NB2, KA2, KB1)

134 (BA1)

135 (LA2, LA, NA1, NB1, KB2)

136 (KA1, KA2, KB1, KB2)

137 (KA1, KA2)

138 (BA2, NA2, NF, KA1)

139 (LA2, LB1, LB2, BA2, BB1, NA1, NB2)

140 (BA1, BA2)

141 (LA2, NB1, KB1)

142 (BB2, BA, NB1, KA1, KB1, KB2)

143 (BB2, KA1, KA2, KB1, KB2, KF)

144 (BA2, NA2, NB1, NB2, KB1)

145 (LB2, NA2)

146 (BA, NA)
